# Value and effectiveness of National Immunization Technical Advisory Groups in low- and middle-income countries: a qualitative study of global and national perspectives

**DOI:** 10.1093/heapol/czz027

**Published:** 2019-05-10

**Authors:** Sadie Bell, Laurence Blanchard, Helen Walls, Sandra Mounier-Jack, Natasha Howard

**Affiliations:** Department of Global Health and Development, London School of Hygiene and Tropical Medicine, 15-17 Tavistock Place, London, UK

**Keywords:** Vaccination, vaccine decision-making, NITAGs, low- and middle-income countries

## Abstract

The Global Vaccine Action Plan proposes that every country establish or have access to a National Immunization Technical Advisory Group (NITAG) by 2020. The NITAG role is to produce evidence-informed recommendations that incorporate local context, to guide national immunization policies and practice. This study aimed to explore the value and effectiveness of NITAGs in low- and middle-income countries (LMICs), identifying areas in which NITAGs may require further support to improve their functionality and potential barriers to global investment. A multi-methods study design was used, comprising 134 semi-structured interviews and 82 literature review sources that included 38 countries. Interviews were conducted with 53 global/regional and 81 country-level participants able to provide insight into NITAG effectiveness, including NITAG members, national immunization programme staff, and global agency representatives (e.g. the World Health Organisation, the Bill and Melinda Gates Foundation, Gavi the Vaccine Alliance). The review, including published and unpublished sources on NITAGs in LMICs, was conducted to supplement and corroborate interview findings. Data were analysed thematically. NITAGs were described as valuable in promoting evidence-informed vaccination decision-making, with NITAG involvement enhancing national immunization programme strength and sustainability. Challenges to NITAG effectiveness included: (1) unreliable funding; (2) insufficient diversity of member expertise; (3) inadequate conflicts of interest management procedures; (4) insufficient capacity to access and use evidence; (5) lack of transparency; and (6) limited integration with national decision-making processes that reduced the recognition and incorporation of NITAG recommendations. LMIC NITAGs have developed significantly in the past decade. Well-functioning NITAGs were trusted national resources that enhanced country ownership of immunization provision. However, many LMIC NITAGs require additional technical and funding support to strengthen quality and effectiveness, while maintaining impartiality and ensuring sufficient integration with national decision-making processes. Barriers to sustainable global support need to be addressed for LMIC NITAGs to both continue and develop further.

**Key Messages**

- Authors drew from 134 global and national-level semi-structured interviews and 82 literature sources that included 38 low- and middle-income countries (LMICs) to provide a broad and robust assessment of perceived National Immunization Technical Advisory Group (NITAG) value, effectiveness and functionality.
- Well-functioning NITAGs appear to be trusted national resources that enhance country ownership of immunization provision, but many LMIC NITAGs require additional support to maintain/strengthen effectiveness.
- If the global community wishes to preserve and strengthen the NITAG decision-making model in LMICs, it needs to recognize existing challenges and mobilize investment to support these country-owned advisory bodies.

## Background

To promote strong and sustainable national immunization programmes, the Global Vaccine Action Plan outlined a 2020 target for all countries to establish or have access to a National Immunization Technical Advisory Group (NITAG) (WHO, 2013). NITAGs are multi-disciplinary bodies of national experts, aiming to provide impartial evidence-based recommendations to guide vaccination decision-making by policy-makers and programme managers (Duclos, 2010). NITAGs are intended to encourage country ownership of immunization programmes by promoting decision-making based on national context, considering factors such as local epidemiology, resource availability, cost-effectiveness and programme sustainability (Duclos, 2010; SIVAC, 2012). Given competing demands on health resources and increasing numbers of new vaccines in low- and middle-income countries (LMICs), the NITAG approach is considered important for determining whether programme recommendations made at global or regional levels are optimal at country-level (Duclos, 2010).

By the end of 2016, 58% of WHO member states (119/194) had a legally or administratively mandated NITAG (WHO/UNICEF, 2018), a 45% increase from 78/194 in 2010 (WHO, 2016a; SAGE, 2017). NITAG performance, as measured by six process indicators included in the WHO/UNICEF Joint Reporting Form (JRF) also improved over time (Duclos, 2010). JRF indicators were: (1) legislative/administrative basis; (2) formal written terms of reference; (3) membership expertise across at least five areas of paediatrics, public health, infectious diseases, epidemiology, immunology, other; (4) at least one meeting annually; (5) agenda and background documents available to NITAG members at least 1 week prior to meetings; and (6) requiring members to declare conflicts of interest. In 2016, 83 NITAGs globally—a 42% increase from 2010 (WHO, 2017)—reported meeting the six JRF indicators.

NITAG progress is particularly notable in LMICs (SAGE, 2017). Between 2010 and 2016, the number of WHO member states reporting a NITAG with a legislative or administrative basis increased from 7% (2/30) to 52% (16/31) in low-income countries and from 41% (43/101) to 61% (67/109) in middle-income countries (SAGE, 2017; WHO/UNICEF, 2018). Similarly, the number reporting a NITAG meeting the six JRF performance criteria increased from 3% (1/30) to 35% (11/31) in low-income countries and from 19% (20/101) to 39% (42/109) in middle-income countries (SAGE, 2017; WHO/UNICEF, 2018).

To support NITAG establishment and strengthening in LMICs, the Bill and Melinda Gates Foundation funded Agence de Médecine Préventive and International Vaccine Institute to implement the *Supporting Independent Immunization and Vaccine Advisory Committees* (SIVAC) Initiative from 2008 to 2017 (AMP, 2018; Howard *et al.*, 2018a; Senouci *et al.*, 2010). SIVAC provided advocacy and partnership development, direct support to NITAG development and strengthening, and indirect NITAG capacity building through materials, publications and tools development (Senouci *et al.*, 2010). This study aimed to explore the value and effectiveness of NITAGs in LMICs, drawing on data from an evaluation of SIVAC support. Objectives were to: (1) describe NITAGs’ role and value, (2) examine elements contributing to NITAG effectiveness, particularly those requiring additional support, and (3) explore potential barriers to global stakeholder investment in NITAGs.

## Methods

### Study design and country eligibility

A qualitative multi-methods study design including semi-structured interviews with global and national informants and a narrative review of published and unpublished literature. Interviews and literature findings were compared during data analysis and interpretation to increase comprehensiveness.

Country eligibility, for national interviews and literature review, was determined by: (1) Gavi eligibility in 2008, or World Bank LMIC status in 2008 plus any reported SIVAC support 2008–17; and (2) JRF reported NITAG in 2016. Thus, among 77 Gavi-eligible and 44 SIVAC-supported LMICs, 55 reported a legally established NITAG in 2016 and were eligible for inclusion.

### Data collection

*Semi-structured interviews* were conducted in 2017 with (1) global/regional and (2) country-level key informants. Global interviewees included members of technical partner agencies, e.g. WHO, the Bill and Melinda Gates Foundation, US-CDC, Gavi, and high-income NITAGs that hosted LMIC NITAG visits. Members of high-income country NITAGs were interviewed about their mentoring and support to specific LMIC NITAGs rather than to discuss their own NITAG. Country-level interviewees included NITAG chairpersons, NITAG secretariat, core and ex-officio members (e.g. WHO, UNICEF) and national stakeholders (e.g. immunization programme managers). Interviewees were approached and recruited via email or at international meetings. Interviews were conducted by NH, HW or SMJ in English, French, Russian or Spanish, either face-to-face or by telephone, at times and places selected by interviewees. Interviews lasted approximately 40 minutes, were audio-recorded, and transcribed professionally.

The *literature review*, conducted in early 2017, included eligible LMICs reporting a NITAG in 2016. First, seven databases were searched systematically from January to March: Medline (Ovid); Embase (Ovid); Global Health (Ovid); Social Policy and Practice (Ovid); Dissertations and Theses in UK and Ireland and Theses Global (ProQuest); Global Index Medicus (bvsalud.org); and Virtual Health Library Regional Portal (bvsalud.org). Searches were conducted in French, Portuguese and Spanish on ProQuest and bvsalud.org. Keywords and their synonyms were: NITAG, vaccine, immunization, advisory, expert, technical, committee.

Secondly, references and websites were hand-searched in March. Hand-searched references and websites included the Vaccine special issue on NITAGs [*Vaccine*, vol. 28(Suppl. 1), 2010]; the NITAG Reference Centre website; WHO and IRIS websites; US-Centers for Disease Control and Prevention Morbidity and Mortality Weekly Report website; reference lists of journal articles included in the review; reference list from a SAGE-commissioned NITAG literature review (John, 2010); and sources obtained from interviewees. Table 1 provides full eligibility criteria. Eligible sources were published between 2006 and 2017 (to cover the period of SIVAC activity), in English, French, Portuguese or Spanish (to cover the languages of most publication on NITAGs in LMICs), and were journal articles, evaluation reports, PowerPoint presentations or administrative forms (e.g. legal documents, operating procedures).

**Table 1.** Literature review eligibility criteria

Criteria	Included	Excluded
Language	English, French, Spanish and Portuguese	Other languages
Publication year	From January 2006 to May 2017	Before 2006 and after May 2017—sources published before 2006 were excluded as: (1) NITAGs were first mentioned in a WHO Regional Technical Advisory Group report on immunization in 2006; (2) only four NITAGs from included countries were established before 2006
Organization	NITAGs	Committees other than NITAGs
Country	LMICs reporting a NITAG in 2016 JRF report	Other countries
Themes	Scope and functions; Relationships with Ministry of Health; Transparency; Conflict of interest; and Capacity to use evidence.	Not about human vaccination and About other themes.
Publication type	Journal articles; Conference abstracts; Evaluation reports from any organization; Descriptive reports from any organization (excluding meeting reports); Presentations (e.g. PowerPoint); NITAG procedures, policies, decrees, nominations, member lists mentioning professions and activity reports; Governmental reports and plans, e.g. NITAG procedures, policies, decrees, nominations, member lists mentioning professions; and SAGE minutes.	Meeting agendas, attendance sheets, minutes; NITAG agreements, work-plans, financial documents, training materials, news, newsletters; Mission reports, workshop reports, study tour reports; Nominations, allocutions, member lists not mentioning professions; NITAG meeting minutes; and NITAG recommendations.

Duplicates were removed and titles, abstracts and eligible full texts screened using EPPI-Reviewer 4 (v4.6.4.0). Screening was performed by LB, with NH double-screening 5% of titles and abstracts (*N* = 82) and SB double-screening 33% of full texts (*N* = 70). Discrepancies were below 5% and resolved by author discussion. Double-screening of a proportion of sources was conducted to ensure rigour while maintaining timeliness and efficiency, as this was a narrative rather than systematic review and inclusion criteria were relatively straightforward (i.e. sources discussed NITAGs in included countries).

### Analysis

Interview data were analysed thematically by NH, SB, HW and SMJ, supported by NVivo version 11, using the stages outlined by Braun and Clarke (2006). Authors used an abductive approach, with deductive codes taken from the interview guide and inductive codes coming from transcript data. Deductive and inductive themes were: (1) NITAG role and value; (2) NITAG functionality/effectiveness; (3) NITAG challenges and enablers; and (4) NITAG achievements.

Literature data were independently extracted by LB and SB, using a 23-category framework developed from the SIVAC tool for evaluating NITAGs (HPID, 2016) plus publication year, type, authors’ affiliations and country described. Two authors synthesized data narratively, including descriptive statistics and illustrative quotes, into five themes: (1) NITAG scope and functions; (2) NITAG management of conflicts of interest; (3) NITAG capacity to use evidence; (4) NITAG transparency; and (5) NITAG linkages with the Ministry of Health. Discrepancies were resolved through author discussion.

## Results

### Scope and themes

Table 2 shows 134 interviews were conducted, 53 global/regional and 81 national. Global interviewees included members of technical partner and donor agencies, e.g. WHO, the Bill and Melinda Gates Foundation. National interviewees, including NITAG and national immunization programme representatives, were recruited from 24 countries across five WHO regions (i.e. WHO Office of the Americas region interviews were not organized before study completion).

**Table 2.** Interviewees and literature sources, by global organization and/or country

Organizational affiliations (*n* = 28) of 53 global interviewees	WHO regions (*n* = 6)	Countries (*n* = 38)^a^	Interviewees by country (*n* = 81)	Literature sources by country (*n* = 85)^e^
AMP (Agence de Médecine Préventive)	Regional Office for Africa (AFRO)	Benin	2	4
AMP-SIVAC (Supporting Independent Immunization and Vaccine Advisory Committees) Initiative		Burkina Faso	2	3
		Cote d'Ivoire	3	11
		Ethiopia	2	0
BMGF (Bill and Melinda Gates Foundation)		Kenya	0	1
		Mali	0	1
ECDC (European Centre for Disease Control)		Mozambique^b^	2	7
		Nigeria	12	5
Gavi, the Vaccine Alliance		Senegal	9	10
Global Health Task Force		Tanzania	1	0
NICE international		Togo	0	1
MOH-Ghana		Uganda	9	7
NITAG-Australia		Zambia	1	0
NITAG-Belgium		Zimbabwe	2	0
NITAG-Canada	Regional Office for the Americas (PAHO)	Honduras	0	3
NITAG-France		Nicaragua^c^	0	1
NITAG-Germany				
NITAG-Netherlands	Regional Office for the Eastern Mediterranean (EMRO)	Pakistan	3	0
NITAG-UK		Tunisia^d^	2	6
NITAG-USA				
PROVAC (Promotion of Evidence-Based Decision-Making on New Vaccine Introductions)				
	Regional Office for Europe (EURO)	Albania	2	0
		Armenia	5	6
US-CDC (US-Centers for Disease Control and Prevention)		Kazakhstan^d^	1	4
		Kyrgyzstan	0	3
VENICE (Vaccine European New Integrated Collaboration Effort)	Regional Office for South East Asia (SEARO)	Moldova	0	2
		Bangladesh	0	2
WAHO (West African Health Organisation)		Bhutan	0	1
WHO-Headquarters		India	2	14
WHO-AFRO		Indonesia	13	8
WHO-AFRO/RITAG (Regional Immunisation Technical Advisory Group)		Myanmar	2	1
		Nepal	1	5
WHO-ESA (Eastern and Southern Africa sub-region)		Sri Lanka	1	4
		Timor-Leste	1	2
WHO-EURO	Regional Office for the Western Pacific (WPRO)	China	1	4
WHO-PAHO		Cambodia^c^	0	2
WHO-SEARO		Lao People’s Democratic Republic	0	1
WHO-WPRO		Mongolia	0	1
		Philippines^d^	2	2
		Viet Nam	0	1

a Seventeen additional eligible countries (i.e. Afghanistan, Azerbaijan, Bosnia and Herzegovina, Cameroon, Cuba, Democratic Republic of the Congo, Djibouti, Georgia, Malawi, Mauritania, Niger, South Sudan, Sudan, Tajikistan, Ukraine, Uzbekistan, Yemen) reported NITAGs in 2016, but no country-level interviews could be conducted or literature sources located.

b Mozambique was incorrectly listed as not having a NITAG in the 2016 JRF, so still included in findings.

c Literature for Cambodia and Nicaragua described quasi-NITAGs and were not included in findings.

d Not Gavi-eligible in 2008, but received SIVAC support.

e Some sources cover multiple countries, so the total does not equal 82.

**Figure 1.**
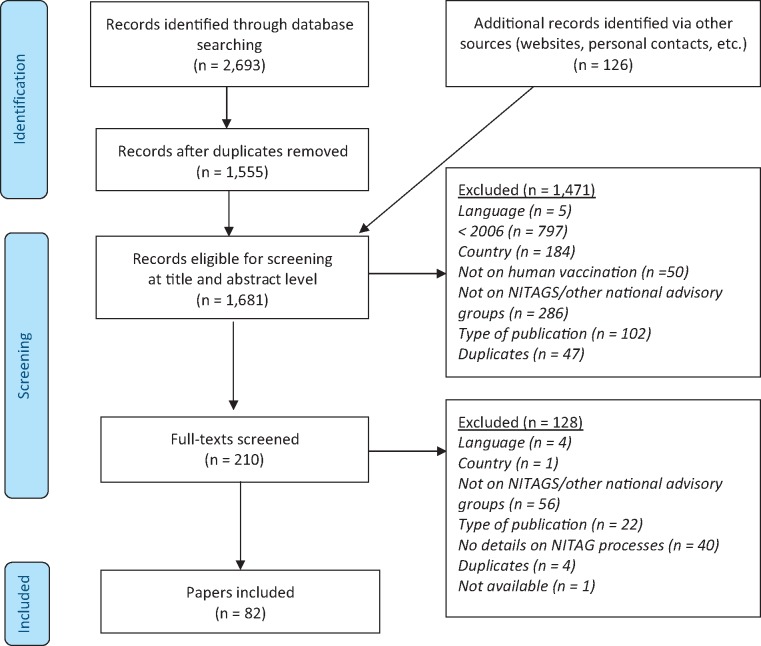
PRISMA flow diagram of literature search.

Figure 1 shows 82 literature sources were identified, referring to NITAGs in 31 countries. Only 13% were empirical research or NITAG evaluations, with the remainder descriptive, e.g. opinion articles and administrative documents. Most (80%) were authored by NITAG members or government entities.

The two overarching themes explored below are: (1) perceived NITAG role and value; and (2) NITAG effectiveness and key perceived challenges. Funding security, identified as the primary challenge to the existence and effectiveness of LIMC NITAGs, is explored further in Howard *et al.* (2018a).

### Perceived NITAG role and value

Interviewees described NITAGs’ role as promoting vaccination policy and programme decision-making based on evidence, rather than opinions or lobbying pressure. Although the NITAG role was most often described as providing guidance on applying global and regional recommendations at country-level, extended NITAG roles were also reported. These included direct involvement in the investigation of vaccination-related adverse events, providing disease prevalence and vaccination surveillance data, and indirect involvement in bio-safety and human resources. NITAG decision-making sometimes went beyond new vaccines, such as NITAGs working to improve national immunization programme credibility and public confidence. For example, an interviewee described how a NITAG’s recommendation was actively disseminated to address vaccine hesitancy and concerns raised by anti-vaccine activists. In the longer term, many NITAG members indicated that revising and broadening the scope of the NITAG was preferable to disbanding as they still saw a valuable role for NITAGs.

*The role of NITAGs should not be limited to the consideration of recommendations for the introduction of new vaccines, but should extend to devising strategies for optimizing the use of existing vaccines and strengthening national immunization programmes* (Country 25-1).

Literature sources described several NITAG as having additional roles and varying degrees of involvement in implementation and operations, from the development of guidance on practical aspects of vaccine introduction (Ba-Nguz *et al.*, 2017) and cold chains (Molina-Aguilera *et al.*, 2010), to addressing ‘bottlenecks’ in programme delivery (John, 2010). Additionally, some NITAGs were involved in research (Ba-Nguz *et al.*, 2017; John, 2010; Molina-Aguilera *et al.*, 2010). The role of the Sri Lankan NITAG was exceptionally broad, covering all communicable diseases, not just those that are vaccine-preventable (Wijesinghe *et al.*, 2010). This NITAG had unusual decision-making power, including revising the national immunization schedule, approving funding mobilization and introducing new vaccines (Wijesinghe *et al.*, 2010).

Interviewees were asked specifically about the value of NITAGs. Many reported that there had been a degree of scepticism towards NITAGs in the early years of SIVAC support, in part due to concerns that NITAGs might delay vaccine introduction. Over time, however, NITAGs were increasingly regarded by the majority of interviewees as essential for effective vaccination policy and programme decision-making, as they became more embedded in national policy-making processes and providing independent, evidence-based recommendations.

*The role of the NITAG is very important. For example, the ministry made the decision to introduce the HPV vaccine but without involving the NITAG. The introduction did not go well and there were major issues of acceptability and resistance, ending up with only two regions with a low coverage rate. There were a lot of negative comments on the vaccine. After that the Ministry of Health arranged for the NITAG to play a bigger and more substantive role* (Country 15-1).

Without NITAGs, interviewees noted that decision-making was not necessarily evidence-based and vaccination programmes were more likely to be sub-optimal, unsustainable and open to undue influence or even corruption.

*…prior to NITAG formation in many countries globally, particularly middle and low-income countries and I would argue even, in the olden days, some high-income countries too, many of those decisions were driven by manufacturers and not driven by evidence, by local vaccine preventable disease data, not driven by how the healthcare system was set up…* (Global 10).

### NITAG effectiveness

NITAG members generally described their NITAG as well-functioning, although several interviewees noted that self-reported JRF functionality criteria were insufficient to fully capture NITAG effectiveness. SIVAC (2016a) describes NITAG effectiveness as encompassing functionality, quality, and integration and all three aspects were deemed important by interviewees.

*They [NITAGs] have to be independent but they also have to have the expertise to do all the work. So, if they are just independent but don’t have the expertise to do the quality, they don’t have the structures to do the quality, it doesn’t matter […] So, you need function; you need quality, and you need integration. You need all three of these…* (Global 10).

Interviewees reported that substantial input was required to ensure effectiveness after NITAG establishment.

*Setting up NITAGs should be a long-term enterprise as it involves developing a culture of evidence-based policy over many years. In high-income countries, this has taken many decades and we should not expect any differently from low-income countries. It is not a two-year project, but rather a 20-year investment.* (Global 12).

NITAG members described several support mechanisms that improved their effectiveness, including capacity-building trainings and access to relevant materials. Additionally, they described visits to other NITAGs—especially to well-established NITAGs in high-income countries—as particularly beneficial to learn about NITAGs’ role and functionality, increase motivation and instil pride in their work.

*[the NITAG] Chairman and other members visited a well-functioning NITAG, […] and before that, as me, they don’t realise what is a NITAG. They didn’t even recognise their role in the NITAG, and it helped, especially for the Chair of the NITAG. Now she’s very proud that she is Head of the NITAG, yeah, [after] experiencing the well-functioning NITAG* (Country 2-3).

Effectiveness was reported as varying depending on context, NITAG maturity, and the type of support received. Key areas discussed as potential challenges included: (1) membership; (2) conflicts of interest; (3) transparency; (4) capacity to gather and use evidence; (5) national recognition and integration within decision-making bodies; and (6) funding security.

### Expertise and diversity of NITAG members

Member expertise and diversity affect NITAG functional capacity. NITAGs aimed to be representative, at least in terms of member expertise and, sometimes other factors, e.g. geographical diversity. Interviewees noted this as challenging for NITAGs in smaller countries with limited numbers of experts and suggested an alternative could be sharing a NITAG between several countries.

*…. some countries are probably too small to have a NITAG, by which I mean finding an adequate number of external experts might be very difficult. In which case it’s possible that countries would need to, several small countries would need to band together to have a NITAG* (Global 42).

The most frequently mentioned area of missing expertise for NITAGs was health economics. The literature revealed that less than half of 24 NITAGs reporting this information had a member with economic expertise. While economic expertise is not included in JRF criteria, many NITAG members indicated its importance. A few NITAGs that lacked economists as members sought external support (Molina-Aguilera *et al.*, 2010; Wijesinghe *et al.*, 2010), an approach recommended by Duclos (2010).

Secretariat capacity was another issue that significantly affected the quality of NITAG processes. Interviewees discussed the importance of a strong secretariat to provide support. For example, NITAGs with only one secretariat member, even a very capable one, reported struggling to ensure functionality and manage workload.

*…. a strong secretariat, meaning it should be well staffed and, in our view…. having more than one person full-time, but secondly, that we should have a mix of skill-sets in the Secretariat…* (Country 41-1).

### Conflict of interest processes

A key feature of NITAG functional capacity is a formal conflict of interest policy with robust procedures to ensure independence from government and other parties with specific agendas, such as vaccine manufacturers.

*[The NITAG] role is extremely important because they need to be totally independent and specifically from the conflicts of interest, particularly from the big pharma […] at country level* (Global 14).

Interviewees highlighted the need for NITAGs to report and address conflicts of interests, although not all NITAGs had adopted formalized conflict of interest policies.

*We are revising the terms of reference, introducing conflict of interest because in the past terms of reference, there was just a statement that conflict of interest should be applied, but actually it was not done* (Country 2-2).

Literature sources identified conflict of interest information for 16 NITAGs, with all but one reporting some form of conflict of interest policy or procedures. However, conflict of interest importance and processes were not universally understood. For instance, reticence to implement conflict of interest policies was noted in Nepal (Schmitt & Batmunkh, 2014), while the Sri Lankan NITAG described the rationale for conflict of interest reporting as public transparency rather than independence of decision-making (Wijesinghe *et al.*, 2010). NITAG members were generally requested to declare any conflict of interest at the start of meetings, and several had processes to address conflicts of interest when declared [Ministry of Health (Mali), 2014; National Primary Health Care Development Agency (NPHCDA) (Nigeria), 2015]. However, information on implementation was scarce. For example, Cote d’Ivoire reported using declaration forms ‘when necessary’ (Benie Bi, 2015), while in Honduras members with conflicts of interest were temporarily suspended and prohibited from voting (Molina-Aguilera *et al.*, 2010). Literature sources were critical of interest groups attempting to influence NITAG decision-making, such as medical associations (Molina-Aguilera *et al.*, 2010). For example, Gavi’s promotion of HPV vaccination in India was described as: ‘…interest groups attempting to push all available vaccines into the national programmes regardless of needs, practical and cost-effectiveness considerations’ (Jayakrishnan, 2013).

For several NITAGs, interviewees and literature sources noted that scarcity of experts could sometimes make it difficult to avoid including any NITAG members engaged at some level with the vaccine industry, e.g. serving as board members (Schmitt and Batmunkh, 2014). Conversely, some interviewees noted that conflicts of interest could be managed effectively by using the appropriate procedures and NITAGs could also be a force for independence, with one NITAG member describing how their country resisted Ministry of Health pressure to procure a less cost-effective vaccine.

### Capacity to gather and use evidence

Though crucial for the quality of NITAG processes, the capacity to collect, synthesize and interpret scientific evidence reportedly varied considerably. Interviewees noted that NITAG members often did not have sufficient time to engage thoroughly with evidence collection, review and interpretation. Additionally, some NITAG members reported difficulties in accessing literature, with paywalls and publication language reported as barriers—a finding also noted in country evaluation literature (SIVAC, 2016a).

*It is not right to assume that because they are experts in their areas, then they know enough to continue to effectively function as NITAG members. They need capacity building and facilitating access to the right information in immunisation, so that then they are in a better position to guide the Ministries of Health. This should be an important part of our work of establishing NITAGs, to continually build the capacity of these people and provide updated information for them to function properly* (Global 17).

Several interviewees commented that more training of NITAG members was needed to help them collect and evaluate evidence.

*I think one of the problems that all NITAGs suffer with is assembly of evidence and so I think that’s probably one of the important areas to have support* (Global 43).

Little documented evidence was found of NITAG literature searching and use, despite this process being essential to NITAGs’ role. Of nine NITAGs for which some data were available, two appeared to conduct structured literature searches (Zheng *et al.*, 2010; NITAG, 2015), three appeared to have detailed standardized processes to analyse evidence and develop recommendations (Ba-Nguz *et al.*, 2017; NITAG, 2015; Uganda National Academy of Sciences, 2016), and four appeared to miss one or more crucial step (Molina-Aguilera *et al.*, 2010; SIVAC, 2016b, 2016c; Wijesinghe *et al.*, 2010). Interviews indicated a preference for using local data and the need to make this readily accessible to NITAGs.

*If we don’t find any* [local data]*, we use the regional data. If there were not any regional data, we use the global data or something like that. So, the important thing is, if possible we use first local, regional and global* (Country 14-16).

However, both interviewees and literature sources noted that local data might not be available. Of 11 countries describing access in literature sources, 7 reported access and 4 reported limited access. Members typically used their affiliation with universities and hospitals to source and report local evidence or relied on links with local partners (Ba-Nguz *et al.*, 2017; Makinen *et al.*, 2012; Wijesinghe *et al.*, 2010). Access to national economic evaluations varied. Of nine NITAGs reporting access information, four sometimes used national economic evaluations (Makinen *et al.*, 2012; Wijesinghe *et al.*, 2010) and five had limited or no access to them (Durupt, 2015; Makinen *et al.*, 2012; SIVAC, 2016a).

Literature sources provided information on type of data used for 12 NITAGs. Epidemiology, disease burden, economic evaluation and WHO recommendations data were most commonly reported, followed by affordability and financial sustainability, vaccine availability and supply, and clinical characteristics or vaccine effectiveness. A few mentioned socio-economic, cultural and equity considerations or recommendations by other NITAGs. While sources were unclear about how NITAGs incorporated social and equity considerations into recommendations, they indicated NITAG decision-making was informed by more than clinical data.

### Transparency

Transparency can improve public trust in vaccination programmes and integration with national decision-making. Literature sources advocate transparency approaches such as open meetings and publishing meeting minutes and recommendations (Duclos, 2010), although interviewees and literature indicated this was not always implemented. Interviewees discussed the importance of ensuring that NITAG processes were transparent, to instil greater public and professional confidence in vaccination programmes.

*In large part, but not all, there has grown the suspicion that there may be collusion links between private interest and a decision taken. It is even more important than ever that the decision and the expertise are separated and that’s something that I think we have achieved in many EU countries. This is one of the most important reasons to have a well-functioning NITAG, not only because a decision will be the right one, but also because it will be perceived as such by the health professionals and by the population…* (Global 38).

For the 12 NITAGs for which access to NITAG meetings was reported, literature sources indicated that most permitted observation by non-members—primarily external experts—upon request (SIVAC, 2016b; Wijesinghe *et al.*, 2010) or by invitation [Ministry of Health (Cote d'Ivoire), 2009; John, 2010; Molina-Aguilera *et al.*, 2010; Wijesinghe *et al.*, 2010; Ministry of Health (Mongolia), 2011; Ministry of Health (Kazakhstan), 2012; Schmitt & Batmunkh, 2014; Uganda National Academy of Sciences, 2014; NPHCDA (Nigeria), 2015; SIVAC, 2016b; Hadinegoro *et al.*, 2017]. Although external attendance was possible in principle, it was unclear how frequently access to meetings by non-members occurred and whether external parties were aware of the possibility of attending meetings.

For the 14 NITAGs for which access to minutes or recommendations were reported, literature sources indicated most were inaccessible to the public [Molina-Aguilera *et al.*, 2010; Wijesinghe *et al.*, 2010; Zheng *et al.*, 2010; Ministry of Health (Kazakhstan), 2012; Schmitt & Batmunkh, 2014; Uganda National Academy of Sciences, 2014; NPHCDA (Nigeria), 2015; Durupt, 2016; SIVAC, 2016c; Hadinegoro *et al.*, 2017]. Literature sources only mentioned two NITAGs routinely providing publicly available minutes and recommendations (John, 2010), while another reported meeting minutes as available upon request (SIVAC, 2016b). In practice, documents described as open access were often unavailable (e.g. not on websites, inaccessible due to broken links, only available for certain meetings or years) or difficult to locate. Some NITAG reports were described as only disseminated by the Ministry of Health (Zheng *et al.*, 2010) or ‘made public after Ministry of Health approbation’ (Nepal National Committee on Immunization Practices, 2010), which potentially limits transparency, e.g. if the Ministry of Health did not agree with NITAG recommendations.

### Integration with national decision-making

Acknowledgement of NITAGs’ role and contributions by national influencers and interaction with decision-makers were considered crucial to NITAG integration within decision-making processes and overall effectiveness (MacDonald *et al.*, 2017). Acknowledgement included awareness of NITAGs’ role, usage of member’s expertise, and implementation and dissemination of NITAG recommendations. Most NITAGs achieved a good level of Ministry of Health recognition, as reported by both NITAG and Ministry of Health interviewees.

*Many members of the NITAG have been invited in several meetings* [at the Ministry of Health…]*. So, I think it’s a very good achievement for them, because their performance is being recognised* (Country 14-23).

However, interviewees reported that NITAG interactions with decision-makers could often be hindered by lack of integration with government, potentially lessening likely adoption of NITAG recommendations. In some cases, NITAG efforts to maintain independence from government prevented the links necessary to ensure adoption of recommendations.

*It can't be totally independent. The independence should come about from… ‘My recommendation for this vaccine is not based on my connection with a pharmaceutical company, or any money that I'm going to make from it,’ but it doesn't mean that it has to be dissociated from the government* (Global 31).

Literature sources identified a broad range of approaches to NITAG communication with government, from embedding NITAGs within government structures (Wijesinghe *et al.*, 2010) to having no government NITAG members or formal relationship with government (SIVAC, 2016b). However, many NITAGs reported having Ministry of Health or government representatives as members. While Duclos and others recommend that for policy support Ministry of Health representatives be ex-officio members without decision-making powers (Duclos, 2010), this was often not the case. For example, the Sri Lankan NITAG was chaired by a government official (Wijesinghe *et al.*, 2010). Government decrees and members did not automatically imply formal communications and interactions. Four NITAGs did not disseminate recommendations directly to Ministry of Health, either because recommendations were listed in minutes with no recommendation document prepared [Nepal National Committee on Immunization Practices (NCIP), 2010; Ministry of Health (Kyrgyzstan), 2012; Durupt, 2015; Durupt, 2016], or because recommendations were submitted via an intermediate (Durupt, 2015).

Interviewees and literature sources indicated that NITAGs need independence from government while maintaining a formal relationship (Duclos, 2010). This tension between independence and integration was described by interviewees as a careful balance of ensuring NITAGs had sufficient government links to be relevant while maintaining independence from government influence in producing recommendations.

*….[the NITAG] can't be totally independent. The independence should come about from… my recommendation for this vaccine is not based on my connection with a pharmaceutical company, or any money that I'm going to make from it. But it doesn't mean that it has to be dissociated from the government* (Global 31).

Interviewees reported that although health ministries broadly adopted NITAG recommendations, implementation delays often occurred, particularly if NITAG deliberations did not examine cost-effectiveness or other policy concerns took precedence.

*We have delays in the implementation of our recommendations because often there are issue of affordability for the government. The introduction of this vaccine has gone up the list—we have a list of priority vaccines to be introduced when finance allows. PCV is at the top, then pertussis for pregnant woman, then Rota and HPV* (Country 40-2).

For example, while it was difficult to assess the timing of recommendations in relation to Gavi grant applications, interviewees indicated that Gavi vaccine introductions were not always reviewed by NITAGs. This seemed particularly the case for vaccines requiring a rapid decision (e.g. Gavi funding ending for transitioning countries).

Several NITAGs reported producing a high number of adopted recommendations (e.g. Mozambique, Sri Lanka) and interviewees indicated governments were increasingly requesting NITAG recommendations, e.g. in response to emerging disease threats or outbreaks. For example, Mozambique recommended implementation of cholera vaccination. Interviewees described NITAGs influencing national policies, with several highlighting that recommendations were adapted to local context (e.g. birth-dose HepB; dengue; RTS, S) in terms of schedule, target population, or programmatic and financial realities.

*….the country was going to introduce probably the* [Merck] *vaccine that’s three doses, and then when they did it, a NITAG review changed it to the two-dose …* [GSK] *vaccine* (Global 33).

NITAG members’ capacity to leverage individual affiliations (e.g. medical associations, private providers) and personal connections with important stakeholders (e.g. policy-makers, influencers) added to NITAGs’ potential influence.

### Further global investment

The most significant challenge to NITAG establishment and strengthening raised by interviewees was the ending of SIVAC’s support, described by almost all interviewees as crucial to LMIC NITAG development and strengthening. A related challenge was access to sustainable funding and technical support, as no global support mechanism was immediately apparent as SIVAC funding ended (Howard *et al.*, 2018a). Most interviewees considered that at least some financing should come from national governments, though independence of NITAG decision-making must be ensured.

The reasons given why the global community had so far failed to fill this gap, despite expressed need by both global and national stakeholders, varied from lack of interest/awareness to other priorities to scepticism. Some interviewees suggested that global partners were not equally committed to NITAGs and that without SIVAC’s momentum NITAGs might ‘fall under the radar’ of key global agencies. Global interviewees suggested various reasons for this. Several criticized SIVAC for not planning post grant, e.g. by developing more sustainable partnerships and making SIVAC’s and NITAGs’ achievements more visible. Some described the persistent scepticism of a few influential global actors regarding the value of NITAGs in low-income countries and ‘lip service’ paid by many in the international community to country ownership (e.g. as country processes might delay or derail vaccine introduction timelines). Others raised concerns about the uneven perceived performance of existing NITAGs, in terms of the length of time they might continue to require external support. A few discussed perceived confusion between regional and national technical advisory groups for immunization and ongoing lack of role clarity, e.g. about which body should be doing what and which was the best body for global partners to support.

## Discussion

Despite limitations, much progress has been achieved by LMIC NITAGs in the past 10 years of global health community support (SAGE, 2017). NITAGs appear unique in being independent yet formal expert committees that are integrated to varying degrees within government decision-making, an innovative approach compared with other vertical disease programmes (e.g. HIV country co-ordinating mechanisms, Ministry of Health linked technical sub-committees for tuberculosis and malaria). This study provides a comprehensive exploration of the value and effectiveness of LMIC NITAGs, and is the first to include such a broad sample of national, regional and global perspectives.

These findings support previous research showing NITAGs are valued for delivering independent evidence-based recommendations to guide national vaccination decision-making (Duclos, 2010; SAGE, 2017; World Health Assembly, 2017). Findings indicate NITAGs were particularly valued for their capacity to strengthen country ownership and inspire public confidence. NITAGs’ primary role involves reviewing the evidence base on vaccines, assessing local data and adapting global or regional recommendations to national contexts. NITAGs’ scope varies, reflecting national history, degree of sophistication and capacity—potentially extending from recommending new vaccines and schedules to roles in monitoring and evaluating immunization safety and programme performance.

Better integrating NITAGs into Ministry of Health decision-making is critical to NITAG relevance, and findings indicated variability related to country context, governance and NITAG capacity to achieve Ministry of Health recognition. Duclos and colleagues recommend that NITAGs have a direct link with senior Ministry of Health officials (Duclos, 2014). However, specific guidance is lacking on how to establish and maintain links, with NITAGs adopting various approaches (MacDonald *et al.*, 2017). SAGE has thus called for developing best practice guidelines (SAGE, 2017). Particularly in countries with higher-functioning NITAGs, Ministries of Health valued the NITAG’s role and independence, suggesting its scope could extend to broader oversight of the immunization programme and, in some cases, using its independent expertise as a public voice for programmes. However, this could create tensions with other bodies, e.g. immunization inter-agency co-ordinating committees that organize funding and implementation.

Determining NITAG effectiveness remains challenging, with both interviewees and literature sources acknowledging the limitations of JRF performance indicators. JRF indicators are useful during NITAG setup, or as a snapshot of progress, and could be adapted to provide better granularity for monitoring functionality alongside, or complemented by, systematic evaluation exercises to pinpoint specific areas for improvement (HPID, 2016). Interviewees repeatedly noted that the development process for these new bodies would take time, with effectiveness and gravitas improving with maturity.

Determining NITAG policy impact is particularly challenging. For example, simply measuring available numbers of recommendations could be deceptive, as recommendations are not always adopted by policy-makers, while their absence could be due, conversely, to systematic review and evidence-based decision-making. However, concurrent in-depth work in five countries (Howard *et al.*, 2018b) provided some convincing examples of evidence-based recommendations that were translated into tailored country-level policy, along with Ministry of Health requests for NITAG recommendations and contributions. Nevertheless, it is important to note that immunization policy decision-making remains complex, and evidence-based components should be understood as one aspect of broader political processes (Burchett *et al.*, 2012). Thus impact is difficult to evaluate.

Many challenges still face recently established LMIC NITAGs, potentially reducing their effectiveness. These include standard operating procedures requiring further strengthening (i.e. conflict of interest management, transparency), understaffed secretariats, member expertise gaps—especially health economics and cost-effectiveness review processes, and ongoing funding uncertainties. Although significant progress has been made, findings showed many NITAGs—notably those more recently established—remained fragile and required further support to achieve the 2020 WHO Global Vaccine Action Plan target (Howard *et al.*, 2018a).

Vaccination programme and NITAG strengthening is particularly crucial in LMICs, as these countries experience the greatest burden of vaccine-preventable disease (WHO, 2013), the lowest reported levels of vaccination coverage (WHO, 2013), large inequalities in vaccination access (Restrepo-Méndez *et al.*, 2016; WHO, 2016b), and long delays in the introduction of new vaccines (WHO, 2013). Thus, interviewees overwhelmingly argued for ongoing global technical and financial support to LMIC NITAGs (Howard *et al.*, 2018a), some highlighting the need to prioritize countries transitioning from Gavi funding while others argued for smaller and lower-income countries (WHO, 2016a). The barriers within the global community, noted by some interviewees, will need to be addressed if sustainable global funding is to be mobilized.

Support could take many forms. Small countries might particularly benefit from collaborating or combining with neighbouring NITAGs to facilitate sharing of expertise (SAGE, 2017). Relatedly, interviewees highlighted the benefits of visits between NITAGs and the potential for broader knowledge-sharing efforts through the Global NITAG Network. Many also advocated for more and better resources, including strengthening NITAG Resource Centre (http://www.nitag-resource.org/) capacity to facilitate access to technical materials and encourage sharing of information and best practices.

Possible study limitations should be considered. These include potential interviewee or source bias, such as NITAG members reporting on their committee’s effectiveness, although the large and diverse sampling frame helped mitigate this. Additionally, although the literature review involved a systematic search, many documents are not routinely shared and/or only produced in national languages, and so not all potential sources could be accessed. The small proportion of titles and abstracts screened in duplicate means that some relevant documents might have been excluded, though screening criteria were straightforward enough that this was unlikely to be a significant issue. Although authors included all eligible countries, only 36 were covered by at least one interview or document, and data for some were quite limited.

## Conclusions

NITAGs have played an increasingly important, albeit disparate, role in LMIC immunization decision-making over the past decade. They are modelled on similar committees in high-income countries that evolved over the past 50 years. Stakeholders at global, regional and national levels recognize the value of NITAGs contributions to evidence-based policy-making, particularly as countries have adopted many new vaccines and vaccination represents a growing national funding requirement. NITAGs face ongoing challenges, and if the global community is to support this decision-making model further, it needs to recognize these and mobilize investment to support the capacity development and strengthening of these country-owned advisory bodies.

## Ethical approval

The London School of Hygiene and Tropical Medicine Observational Research Ethics Committee granted ethics approval (reference 12036).

## Author contributions

SB, SMJ and NH drafted the manuscript. NH and SMJ conceived the study. NH, HW and SMJ collected interview data. LB conducted literature searches. NH, SB and HW coded and analysed interview data, while LB and SB coded and analysed literature data. All authors approved the version for submission.
